# Suppression of Leukotriene B_4_ Generation by *Ex-vivo* Neutrophils Isolated from Asthma Patients on Dietary Supplementation with Gammalinolenic Acid-containing Borage Oil: Possible Implication in Asthma

**DOI:** 10.1080/10446670410001670445

**Published:** 2004-03

**Authors:** Vincent A. Ziboh, Stanley Naguwa, Kao Vang, Julie Wineinger, Brian M. Morrissey, J. A. McIntyre, Mitchell Watnik, M. Eric Gershwin

**Affiliations:** 1 Division of Rheumatology Allergy and Clinical Immunology Departments of Dermatology and Internal Medicine University of California at Davis School of Medicine, TB 192 Davis CA 95616 USA; 2 Division of Pulmonary Medicine Department of Statistics University of California at Davis School of Medicine, TB 192 Davis CA 95616 USA; 3 Division of Rheumatology Allergy and Clinical Immunology Departments of Dermatology and Internal Medicine University of California at Davis School of Medicine, TB 192 Davis CA 95616 USA

## Abstract

Dietary gammalinolenic acid (GLA), a potent inhibitor of 5-lipoxygenase (5-LOX) and suppressor of leukotriene B_4_ (LTB_4_), can attenuate the clinical course of rheumatoid arthritics, with negligible side effects. Since Zileuton, also an inhibitor of 5-LOX, attenuates asthma but with an undesirable side effect, we investigated whether dietary GLA would suppress biosynthesis of PMN-LTB_4_ isolated from asthma patients and attenuate asthma. Twenty-four mild-moderate asthma patients (16–75 years) were randomized to receive either 2.0 g daily GLA (borage oil) or corn oil (placebo) for 12 months. Blood drawn at 3 months intervals was used to prepare sera for fatty acid analysis, PMNs for determining phospholipid fatty acids and for LTB4 generation. Patients were monitored by daily asthma scores, pulmonary function, and exhaled NO. Ingestion of daily GLA (i) increased DGLA (GLA metabolite) in PMN-phospholipids; (ii) increased generation of PMN-15-HETrE (5-LOX metabolite of DGLA). Increased PMN-DGLA/15-HETrE paralleled the decreased PMN generation of proinflammatory LTB_4_. However, the suppression of PMN-LTB4 did not reveal statistically significant suppression of the asthma scores evaluated. Nonetheless, the study demonstrated dietary fatty acid modulation of endogenous inflammatory mediators without side effects and thus warrant further explorations into the roles of GLA at higher doses, leukotrienes and asthma.


*Clinical & Developmental Immunology*, March 2004, Vol. 11 (1), pp. 13–21

Taylor&Francis healthsciences

### Suppression of Leukotriene $B_4$ Generation by *Ex-vivo* Neutrophils Isolated from Asthma Patients on Dietary Supplementation with Gammalinolenic Acid-containing Borage Oil: Possible Implication in Asthma

VINCENT A. ZIBOH[a,b], STANLEY NAGUWA[a,b], KAO VANG[a,b], JULIE WINEINGER[a,b], BRIAN M. MORRISSEY[a,b], MITCHELL WATNIK[a,b] and M. ERIC GERSHWIN[a,b,*]

[a]*Division of Rheumatology, Allergy and Clinical Immunology, Departments of Dermatology and Internal Medicine, University of California at Davis School of Medicine, TB 192, Davis, CA 95616, USA;* [b]*Division of Pulmonary Medicine, Department of Statistics, University of California at Davis School of Medicine, TB 192, Davis, CA 95616, USA*

Dietary gammalinolenic acid (GLA), a potent inhibitor of 5-lipoxygenase (5-LOX) and suppressor of leukotriene $B_4$ ($LTB_4$), can attenuate the clinical course of rheumatoid arthritics, with negligible side effects. Since Zileuton, also an inhibitor of 5-LOX, attenuates asthma but with an undesirable side effect, we investigated whether dietary GLA would suppress biosynthesis of PMN-$LTB_4$ isolated from asthma patients and attenuate asthma. Twenty-four mild-moderate asthma patients (16–75 years) were randomized to receive either 2.0 g daily GLA (borage oil) or corn oil (placebo) for 12 months. Blood drawn at 3 months intervals was used to prepare sera for fatty acid analysis, PMNs for determining phospholipid fatty acids and for $LTB_4$ generation. Patients were monitored by daily asthma scores, pulmonary function, and exhaled NO. Ingestion of daily GLA (i) increased DGLA (GLA metabolite) in PMN-phospholipids; (ii) increased generation of PMN-15-HETrE (5-LOX metabolite of DGLA). Increased PMN-DGLA/15-HETrE paralleled the decreased PMN generation of proinflammatory $LTB_4$. However, the suppression of PMN-LTB4 did not reveal statistically significant suppression of the asthma scores evaluated. Nonetheless, the study demonstrated dietary fatty acid modulation of endogenous inflammatory mediators without side effects and thus warrant further explorations into the roles of GLA at higher doses, leukotrienes and asthma.

*Keywords*: Dietary gammalinolenic acid; 5-Lipoxygenase; Leukotriene $B_4$; Zileuton

### INTRODUCTION

Gamma-linolenic acid (18:3*n*-6) is relatively present in large amounts in the plant seed oils of borage (18–26% GLA), black currant (15–20%), and evening primrose (7–10% GLA), as well as in fungal oil (23–26%). The triacylglycerol stereospecific position of GLA varies with the source of the oils and this is important in establishing their relative efficacies. For instance, GLA is concentrated in the sn-3 position of the glycerol bridge of the triacylglycerol in evening primrose and black currant seed oil. In contrast, GLA is concentrated in the sn-2 position of the glycerol bridge in borage oils and in the sn-2/sn-3 positions of fungal oil (Lawson and Hughes, 1988).

In both humans and rodents, only a small fraction of the DGLA resulting from the elongation of GLA is converted by the $\Delta 5$ desaturase to arachidonic acid (Stone *et al.*, 1979; Ziboh and Fletcher, 1992; Johnson *et al.*, 1997). This minor conversion is due to the low activity of $\Delta 5$ desaturase in most tissues, thus reducing the concerns that dietary GLA/DGLA would contribute to the excessive accumulation of arachidonic acid and consequently the generation of pro-inflammatory metabolites such as $LTB_4$, $LTC_4$ and $LTD_4$ (Zurier *et al.*, 1996; Johnson *et al.*, 1997). The preceding reports indicate that in many tissues and cell-types, DGLA, but not arachidonic acid, is what accumulates in tissues and cells after GLA supplementation in the diet. The accumulated DGLA, is oxygenated via the cyclooxygenase pathway to prostaglandin of the 1-series ($PGE_1$) and via the 15-lipoxygenase (15-LOX) pathway to 15(*S*)-hydroxyeicosatrienoic acid (15-HETrE)

*Corresponding author. Address: Division of Rheumatology, Allergy and Clinical Immunology, Departments of Dermatology and Internal Medicine, University of California at Davis School of Medicine, TB 192, Davis, CA 95616, USA. Tel.: +1-530-752-2884. Fax: +1-530-752-4669. E-mail: megershwin@ucdavis.edu

ISSN 1740-2522 print/ISSN 1740-2530 online © 2004 Taylor & Francis Ltd
DOI: 10.1080/10446670410001670445

(Borgeat *et al.*, 1976). These two oxidative metabolites of DGLA ($PGE_1$ and 15-HETrE) have been reported to exert biological and clinical effects notably the suppression of acute and chronic inflammation in a variety of systems and disease conditions. In addition, DGLA can compete with arachidonic acid for cyclooxygenase, thus reducing the production of prostaglandin of the 2-series ($PGE_2$). Similarly, DGLA is metabolized by the 15-LOX to 15-HETrE which is a potent inhibitor of the synthesis of the pro-inflammatory mediators $LTB_4$, $LTC_4$ and $LTD_4$ from arachidonic acid via the 5-LOX pathway. Interestingly, 15-HETrE has been shown to markedly inhibit $LTB_4$ generation from arachidonic acid by rat basophilic leukemia (RBL-I) cells *in vitro* (Vanderhoek *et al.*, 1980; Miller *et al.*, 1988). Similarly, hamsters fed GLA-containing oil revealed significantly elevated levels of 15-HETrE *in vivo* in the hamster lung which paralleled greatly reduced $LTB_4$ generation by PMN-infiltrated hamster lung (Ziboh *et al.*, 1997).

Promising therapeutic reports have also emerged after dietary GLA supplementation in patients with rheumatoid arthritis (RA) (Leventhal *et al.*, 1993). Asthma on the other hand is characterized by variable and reversible airflow obstruction and by bronchial hyperresponsiveness, as well as excessive airway narrowing in response to a variety of apparently unrelated stimuli. Although contraction of the airway smooth muscle has been emphasized as an important mechanism of asthmatic airway obstruction, it is now recognized that edema of the airway wall resulting from microvascular leakage, cellular infiltration and luminal obstruction with plasma exudation, cellular debris and airway secretions are all contributory. Inflammation in the airway wall is therefore a prominent feature of fatal asthma attack (Dunnill, 1960; Glynn and Michaels, 1960). There is abundant experimental evidence that inflammation is also present in mild asthma and is related to bronchial hyperresponsiveness (Chung, 1986), a characteristic feature of asthma (Boushey *et al.*, 1980). The pathological changes are therefore likely to include the release of inflammatory mediators such as leukotrienes. Therefore, we began a prospective double blind study designed to address whether dietary supplement GLA alters the leukotriene profile and the clinical readouts of patients with asthma.

### PATIENTS AND METHODS

### Study Design

This is a 12-month, randomized, double-blind comparison of dietary supplementation of gammalinolenic acid (GLA)-containing oil (borage oil) to placebo (corn oil) in patients with asthma. The protocol was reviewed and approved by the Committee for the Protection of Human Subjects in Research at the University of California School of Medicine, Davis. Written informed consent was obtained from each patient. Patients were eligible to participate in the study if their asthma condition satisfied the criteria of steps 2 (mild persistent) and 3 (moderate persistent) of the Expert Panel Report 2: Guidelines for the Diagnosis and Management of Asthma (1997). Approximately 80 adult patients were screened, of which 54 met the criteria for inclusion and whose data is presented herein. These included a total of 11 male and 43 female patients. Of these, the average age of male subjects in the borage group was 52 with a range of 16–71, compared to the average age of male subjects in the control group of 53, with a range of 44–62. The average age of female subjects in the borage group was 42, with a range of 18–70, compared to the average age of female subjects in the control group of 45, with a range of 28–69.

### Dietary Protocol

Patients 16–75 years who were deemed eligible according to the above criteria were randomly assigned to receive either GLA (as borage oil) or placebo (as corn oil). Each borage capsule contained approximately 500 mg of GLA plus 13 IU of D-α-tocopherol (vitamin E) to minimize oxidation. Similar amount of D-α-tocopherol was contained in the corn oil capsule. The total daily dose to each participant (two capsules two times daily with meals) provided 2.0 g of gammalinoleic acid per day. Placebo capsules which contained corn oil were identical in appearance and size and were taken according to the same schedule. The patients were instructed to remain on their regular medications, have serial clinical evaluation, count the number of any returned capsules, and provide blood for isolation of PMNs and fatty acid analysis

### Clinical Protocol

#### *Measurement of $FEV_1$*

At the baseline evaluation, patients underwent complete history and physical examination with particular emphasis on asthma severity based on NAEPP II guidelines. Patients also underwent measurement of FVC, $FEV_1$ and peak flow measurements in addition to being provided the Juniper Quality of Life Questionnaire. Daily wheezing scores and use of rescue medication was also quantitated.

#### *Exhaled NO Assay*

Measurements of exhaled NO was performed using a restricted exhaled breath, off-line technique (Recommendations for Standardized Procedures for the Online and Offline Measurement of Exhaled Lower Respiratory Nitric Oxide and Nasal Nitric Oxide in Adults and Children, 1999) with an effective flow rate of 100 ml/s. NO levels were measured in triplicate for each patient and time point using a chemiluminescence analyzer (Sievers Instruments, Boulder, CO, USA). Patients returned every 3 months for repeat evaluation.

### Analysis of Serum Fatty Acids

Blood was obtained by venipuncture. One portion of the blood was collected in heparinized tubes and used to prepare serum for fatty acids analysis. Typically, each 100 μl serum sample in a conical tube was extracted with 4 ml of $CHCl_3$:$CH_3OH$ (2:1, v/v) and 0.8 ml 0.1 M KCl to obtain total lipids. The mixture was vortexed for 1 min and then centrifuged at 600*g* for 20 min at 4°C. The lower organic phase was collected and a known concentration of heptadecanoate was added as an internal standard and both dried under nitrogen gas. The extracted serum total lipids were transmethylated in 6% methanolic HCl and the resulting fatty acid methyl esters extracted into petroleum ether and dried under nitrogen gas. The residue was dissolved in dichloromethane and injected into a gas-liquid-chromatographic instrument. The gas-chromatographic instrument used (model GC-17A, Shimadzu, Pleasanton, CA) was equipped with a DB-225 fused silica capillary column (50% cyanopropylphenyl, 0.15 mm film thickness, 30 m × 0.25 mm id; J&W Scientific, Rancho Cordova, CA). Hydrogen (36 cm/s) was used as the carrier gas, the oven was run isothermally at 210°C, and the detection was performed with a flame-ionization detector (FID).

### Preparation of PMNs

The other portion of the whole blood obtained by veniculture was collected in vacutainer tubes containing EDTA. Neutrophils were isolated according to the instructions provided by the commercial kit of Robbins Scientific Corp. (Sunnyvale, CA) for the isolation of human neutrophils. Briefly, the EDTA-anticoagulated blood, (3.5 ml) was layered carefully over 3.8 ml of the PMN medium (metrizoate, 13.8%, w/v:dextran, 8.0% w/v) in a 10 ml centrifuge and then centrifuged at 500*g* for 35 min in a swing out rotor at room temperature. After centrifugation the middle band in the upright tube, which comprised mostly of PMNs was removed with pasteur pipette. The removed PMN cells were diluted with equal amount of 0.5 NaCl and then transferred to a 10 ml tube. The tube was filled with 0.9% saline and then centrifuged at 40*g* for 10 min at room temperature to remove any contaminating red cell.

### Analysis of PMN Leukotriene $B_4$

The purified PMNs were resuspended in Hank's balanced-salt solution (HBSS) which contained no calcium chloride, magnesium chloride or magnesium sulfate (Gibco, Grand Island, NY) and then used for assays. Five hundred microliters of the neutrophil suspension ($1 \times 10^7$ cells) in $Ca^{2+}$-free HBSS were added to five-hundred microliter of HBSS buffer containing 1.6 mM $Ca^{2+}$ to bring the final $Ca^{2+}$ concentration to 0.8 mM. The neutrophil suspension was preincubated at 37°C for 10 min. The activation of the cells was initiated by the addition of 5 μl of $Ca^{2+}$ ionophore (A23187) (previously dissolved in DMSO and diluted in $Ca^{2+}$-free HBSS). The final $Ca^{2+}$ ionophore concentration was 2 μM, and the final DMSO concentration was 0.1% (v/v). The mixture was incubated at 37°C for an additional 10 min. The reaction was stopped by rapid cooling on ice at 4°C and immediately followed by centrifugation at 600*g* at 4°C for 10 min. The supernatant was removed and acidified to pH 3.0 with 9% formic acid in a drop-wise manner to precipitate out the proteins. The acidified extract was extracted with 10 ml of $CHCl_3$:$CH_3OH$ (2:1, v/v), and 250 ng prostaglandin $B_2$ ($PGB_2$) was added as an internal standard for quantification of $LTB_4$. The lipid mixture was extracted by vortexing for 1 min and then centrifuged at 600*g* for 10 min at 4°C. The lower organic phase was collected and evaporated in a rota-vapor and the residue suspended in 100% ethanol and stored at −20°C until analyzed by HPLC. The HPLC system used consisted of a Beckman System Gold 125 Solvent Module pump, a 5-μm $C_{18}$ Hypersil® ODS RP-HPLC columns (4.6 × 250 mm) (ThermoHypersil, Bellefonte, PA), and a Beckman Gold System 168 Detector with an on-line diode array scan. Each column was eluted isocratically for 60 min at a flow rate of 1.0 ml/min with the mobile phase of acetonitrile/methanol/water/acetic acid (29:19:52:1 by volume). The pH of the eluting mobile phase was adjusted to 5.6 with 1.0 M NaOH which resolves the leukotrienes. Absorbance was continually monitored at 280 nm during elution. Additional identification of $Ca^{2+}$ ionophore-induced PMN generated products was by characteristic ultraviolet absorbance and comparison with the authentic $LTB_4$ as reported previously (Johnson *et al.*, 1997). Quantitation of generated $LTB_4$ was determined by using authentic standard $LTB_4$ obtained from Cayman Chemicals, Ann Arbor, MI.

### Analysis of PMN Phospholipid Fatty Acids

A portion of the isolated PMNs were extracted with Folch mixture ($CHCl_3$:$CH_3OH$ 2:1, v/v), dried under nitrogen gas and then applied to thin layer chromatographic (TLC) plates coated with Silica Gel G (0.25 mm thickness) (Merck, Darmstadt, Germany). The plates were developed in the solvent system: chloroform/methanol/acetic acid/water 90:8:1:0.8, by volume. The band representing total phospholipids on the TLC plates was first sprayed with 0.2% 2′,7′-dichlorofluorescein in ethanol and then visualized under ultraviolet light. The TLC band which correlated with total phospholipid standard was scraped off the plate and then eluted with chloroform/methanol (2:1, v/v). An internal standard, heptadecanoate, was added to the mixture and again dried. The eluted total phospholipids were transmethylated in 6% methanolic HCl, and the resulting fatty acid methyl esters were extracted with petroleum ether and dried under nitrogen gas. The mixture of fatty acids was separated and quantitated in a Shidmadzu model GC-17A gas chromatograph as previously described for serum fatty acids (Ziboh and Fletcher, 1992).

TABLE I Fatty acid composition of dietary capsules

| Fatty acid | Borage oil (mg/100 mg total fatty acids) | Corn oil (mg/100 mg total fatty acids) |
|---|---|---|
| 16:0 | 6.82 | 9.32 |
| 18:0 | 2.72 | 2.69 |
| 18:1*n*-9 | 10.13 | 25.02 |
| 18:2*n*-6 | 23.13 | 60.92 |
| 18:3*n*-6 (GLA) | 45.86 | – |
| 20:3*n*-6 (DGLA) | – | – |
| 20:4*n*-6 (AA) | – | – |
| 18:3*n*-3 | 0.47 | 1.75 |
| 20:5*n*-3 | – | – |
| 22:6*n*-3 | – | – |

Details of transmethylation and preparations of fatty acid methyl esters are described in the "Method" section. The number after "*n*" indicates the number of carbon atoms from the methyl end of the acyl chain to the nearest double bond. Values are expressed as percentage of total fatty acids for corn oil (100%), or borage oil (100%). The abbreviations represent: GLA, gammalinolenic acid; DGLA, dihomogammalinolenic acid; AA, arachidonic acid.

### RESULTS

### Fatty Acid Profile in the Dietary Borage Oil and Corn Oil

Aliquots of 10 μl from each of the capsules containing either borage oil or corn oil was extracted first with $CHCl_3$:$CH_3OH$ (2:1, v/v) and taken to dryness under nitrogen gas. The transmethylation and preparation of fatty acid methyl esters are as described in the "Method" section. Table I illustrates the fatty acid profiles in the dietary capsules. The major polyunsaturated fatty acid (PUFA) in the borage capsule was GLA (18:3*n*-6) whereas the major PUFA in the placebo (corn oil) was linoleic acid (LA, 18:2*n*-6). Arachidonic acid was negligible in both dietary oils.

### Fatty Acid Profile in the Sera of Patients

The fatty acid profile of both the placebo (corn oil) and the experimental (borage oil) groups are shown in Table II. The data show serum fatty acid profiles at 3 month intervals over the 12 months of the study. There was a statistically significant increase in GLA and DGLA in particular, during the 3, 6 and 12-month evaluations in the patients ingesting GLA-containing borage oil when compared to the placebo (corn oil group). There was a minor but insignificant elevation of AA in both groups over the months of study.

### Fatty Acid Profile in Neutrophil Phospholipids

To determine whether or not the dietary intake of GLA does exert attenuating effect on neutrophil generation of proinflammatory $LTB_4$, we determined the fatty acid profile of the isolated neutrophils as described in the "Methods" section. The data shown in Table III revealed a statistically increased incorporation of GLA/DGLA into total PMN phospholipids of patients who ingested GLA-containing borage oil at the 6th and 12th months were compared to the placebo corn oil group. Of particular note was the statistically significant ($p <$ 0.05) suppression of AA incorporation into the PMN phospholipids of the patients who ingested GLA-containing oil when compared to the placebo corn oil group. This finding is in contrast to the AA profile in the serum and also cautions using plasma/serum fatty acids alone (Table II) as the final determinant of fatty acids levels in the tissues. This finding suggests that whereas there is an active elongase activity in the PMNs that converted GLA to DGLA, there is reduced Δ5 desaturase activity for conversion of DGLA to AA. This is consistent with a previous reported *in vitro* metabolism of GLA in PMN (Chilton *et al.*, 1996). The mechanism of AA suppression in the GLA-fed group is unclear. However, the finding does imply that increased DGLA could result in its metabolism *in vivo* to metabolites that inhibit PMN-induced generation of proinflammatory $LTB_4$.

TABLE II Time-dependent influence of dietary supplimentation with corn oil and borage oil on patients' serum unsaturated fatty acids level

| UFA | Patients | Basal month 0 (mol% ± SEM) | Month 3 (mol% ± SEM) | Month 6 (mol% ± SEM) | Month 12 (mol% ± SEM) |
|---|---|---|---|---|---|
| 18:1*n*-9 | C | 21.1 ± 1.1 | 19.4 ± 0.8 | 21.1 ± 0.6 | 21.2 ± 1.5 |
| | B | 23.3 ± 0.8 | 21.6 ± 0.7 | 20.5 ± 0.8 | 21.5 ± 0.8 |
| 18:2*n*-6 | C | 29.3 ± 1.1 | 32.1 ± 0.8 | 32.1 ± 1.3 | 31.6 ± 2.3 |
| | B | 28.1 ± 1.1 | 27.5 ± 0.8 | 27.9 ± 0.7 | 27.2 ± 0.7 |
| 18:3*n*-6 (GLA) | C | 0.5 ± 0.1 | 0.5 ± 0.1 | 0.5 ± 0.1 | 0.4 ± 0.1 |
| | B | 0.5 ± 0.1 | 1.2* ± 0.1 | 1.1* ± 0.1 | 1.2* ± 0.1 |
| 20:3*n*-6 (DGLA) | C | 1.3 ± 0.1 | 1.4 ± 0.1 | 1.4 ± 0.1 | 1.4 ± 0.2 |
| | B | 1.1 ± 0.1 | 2.4* ± 0.2 | 2.3* ± 0.3 | 2.5* ± 0.2 |
| 20:4*n*-6 (AA) | C | 5.9 ± 0.5 | 6.5 ± 0.4 | 6.6 ± 0.3 | 7.0 ± 0.8 |
| | B | 5.3 ± 0.4 | 6.9 ± 0.4 | 7.0 ± 0.4 | 7.6 ± 0.4 |
| 18:3*n*-3 | C | 0.8 ± 0.1 | 0.7 ± 0.1 | 0.8 ± 0.1 | 0.7 ± 0.1 |
| | B | 0.8 ± 0.1 | 0.7 ± 0.1 | 0.8 ± 0.1 | 0.7 ± 0.1 |
| 20:5*n*-3 | C | 0.8 ± 0.2 | 0.8 ± 0.2 | 0.7 ± 0.2 | 0.6 ± 0.3 |
| | B | 0.5 ± 0.1 | 0.5 ± 0.1 | 0.5 ± 0.1 | 0.5 ± 0.1 |
| 22:6*n*-3 | C | 1.4 ± 0.2 | 1.4 ± 0.2 | 1.4 ± 0.2 | 1.5 ± 0.3 |
| | B | 1.2 ± 0.2 | 1.2 ± 0.2 | 1.3 ± 0.1 | 1.3 ± 0.1 |

Details of transmethylation and preparations of fatty acid methyl esters are described in the "Method" section. The number after "*n*" indicates the number of carbon atoms from the methyl end of the acyl chain to the nearest double bond. Values are expressed as mol% of total serum fatty acids ± SEM. The "number" of patients on C (Corn oil) is 11; The "number" of patients on B (Borage Oil) is 19. Values with * are significantly different ($p < 0.05$).

TABLE III Time-dependent influence of dietary supplementation with corn oil and borage oil on patients' PMN—total phospholipid unsaturated fatty acids level

| UFA | Patients | Basal month 0 (mg/100 mg Total Phospholipid ± SEM) | Month 3 (mg/100 mg Total Phospholipid ± SEM) | Month 6 (mg/100 mg Total Phospholipid ± SEM) | Month 12 (mg/100 mg Total Phospholipid ± SEM) |
|---|---|---|---|---|---|
| 18:1*n*-9 | C | 10.4 ± 1.1 | 10.6 ± 1.1 | 10.1 ± 1.0 | 11.3 ± 1.2 |
| | B | 11.4 ± 1.1 | 10.9 ± 1.0 | 11.2 ± 0.7 | 11.4 ± 0.8 |
| 18:2*n*-6 | C | 4.1 ± 0.5 | 4.5 ± 0.7 | 4.4 ± 1.3 | 4.6 ± 0.7 |
| | B | 4.3 ± 0.6 | 5.0 ± 0.5 | 5.1 ± 0.5 | 4.8 ± 0.5 |
| 18:3*n*-6 (GLA) | C | 1.0 ± 0.1 | 0.9 ± 0.1 | 0.9 ± 0.1 | 0.9 ± 0.1 |
| | B | 0.9 ± 0.1 | 1.1 ± 0.1 | 1.4* ± 0.1 | 1.6* ± 0.1 |
| 20:3*n*-6 (DGLA) | C | 0.9 ± 0.1 | 0.9 ± 0.1 | 0.9 ± 0.1 | 0.9 ± 0.1 |
| | B | 0.7 ± 0.1 | 1.0 ± 0.1 | 1.3* ± 0.1 | 1.4* ± 0.1 |
| 20:4*n*-6 (AA) | C | 5.7 ± 0.4 | 5.5 ± 0.4 | 6.4 ± 0.5 | 6.5 ± 0.5 |
| | B | 5.8 ± 0.4 | 4.5* ± 0.6 | 4.1* ± 0.4 | 4.0* ± 0.4 |
| 18:3*n*-3 | C | Tr | Tr | Tr | Tr |
| | B | Tr | Tr | Tr | Tr |
| 20:5*n*-3 | C | Tr | Tr | Tr | Tr |
| | B | Tr | Tr | Tr | Tr |
| 22:6*n*-3 | C | Tr | Tr | Tr | Tr |
| | B | Tr | Tr | Tr | Tr |

Details of transmethylation and preparations of fatty acid methyl esters are described in the "Method" section. The number after "*n*" indicates the number of carbon atoms from the methyl end of the acyl chain to the nearest double bond. Values are expressed as mg/100 mg of PMN total phospholipid fatty acids ± SEM. Values with * are significantly different ($p < 0.05$). The letter "Tr" indicates trace.

### Dietary GLA Supplementation Suppresses *Ex Vivo* PMN Biosynthesis of $LTB_4$

To delineate whether the increased DGLA in PMNs would exert effect on $Ca^{2+}$ ionophore stimulated isolated PMNs, we incubated the isolated PMNs from both the GLA-supplemented group and the corn oil supplemented group with $Ca^{2+}$ ionophore. Our data, as shown in Fig. 1 revealed a statistically significant ($p < 0.05$) time-dependent suppression of PMN-$LTB_4$ generation by dietary supplementation of GLA-containing borage oil at 6 and 12 months. In contrast, the PMN generation of $LTB_4$ in the placebo corn oil supplemented group revealed negligible alteration (data not shown). This finding does demonstrate that a relationship exists between the length of time of dietary intake of GLA-containing borage oil and suppression of PMN capability to biosynthesize $LTB_4$.

The data in Fig. 2 reveals the relationship of PMN phospholipid DGLA (A) and PMN $LTB_4$ (B) at the 12th month, the end of the dietary study. Specifically after 12 months of dietary borage supplementation, the data in Fig. 2 revealed that increased incorporation of DGLA into PMN phospholipids (Fig. 2A) paralleled the suppression of $Ca^{2+}$ ionophore activated PMN generation of $LTB_4$ (Fig. 2B), indicating more accumulation of DGLA in the PMN exerts its effect via the attenuation of PMN generation of $LTB_4$.

### Clinical Responses

Mixed model analysis of variance (ANOVA) methods were used to model the outcomes of $FEV_1$, peak flow, "wheezing score", and "rescue usage", with the subjects

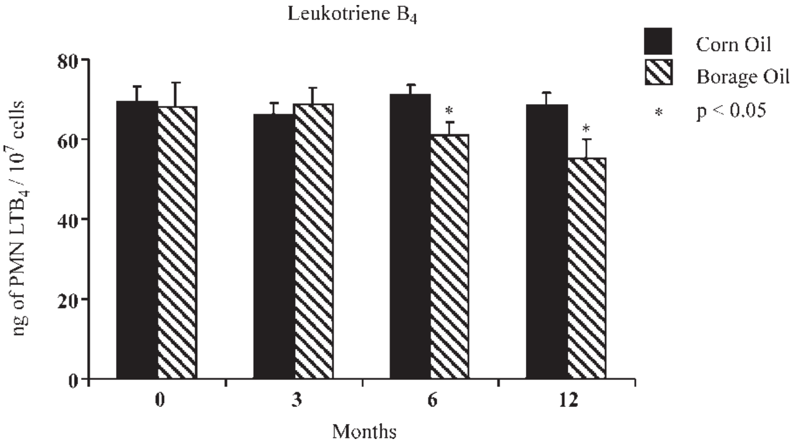

FIGURE 1 Time-dependent effect of dietary GLA supplement on PMN generation of $LTB_4$. Blood from asthma patients whose diets were either supplemented with either 2.0 g GLA/day as borage oil or placebo corn oil were collected respectively in EDTA at 3-monthly intervals. Neutrophils were isolated as described in the "Methods" section and then stimulated with $Ca^{2+}$ ionophore. The extract was subjected to separation on HPLC to identify and quantify for $LTB_4$. Patients whose diets were supplemented with GLA-containing borage oil significantly ($p < 0.05$) suppressed $Ca^{2+}$ ionophore-induced PMN generation of $LTB_4$. Results are means ± SEM. Borage oil group is 19 and corn oil group is 11.

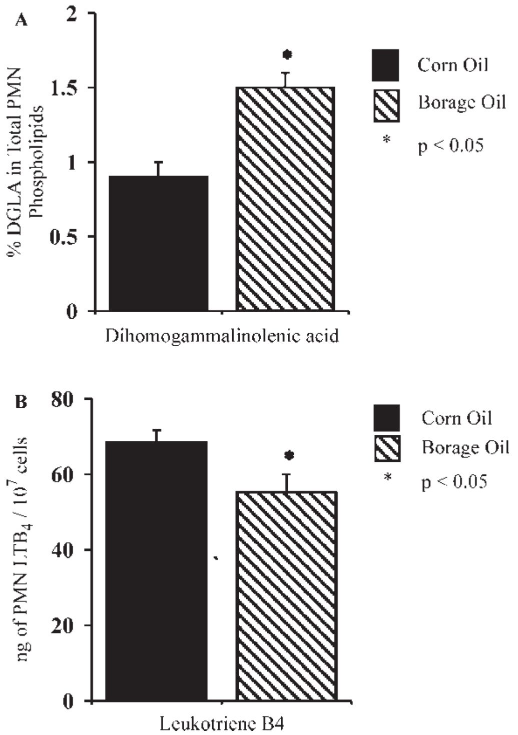

FIGURE 2 Relationship of elevated incorporation of DGLA into PMN total phospholipids and decreased PMN generation of $LTB_4$. Blood from asthma patients whose diets after 12 months were either supplemented with either 2.0 g GLA/day as borage or corn oil were collected, respectively, in EDTA as described in Fig. 1. The procedures for identification of PMN $LTB_4$ are also described in Fig. 1 and PMN-DGLA is as described in Table III. (A) Shows relationship of PMN DGLA in corn and borage oil groups and (B) the relationship of PMN-$LTB_4$ in corn and borage oil groups.

TABLE IV Quantitation of $FEV_1$

| Treatment group | | $FEV_1$ control | $FEV_1$ borage oil |
|---|---|---|---|
| Pre-study | Mean | 0.90 | 0.89 |
| | Min | 0.61 | 0.69 |
| | Median | 0.86 | 0.88 |
| | Max | 1.15 | 1.15 |
| Month 0 | Mean | 0.86 | 0.86 |
| | Min | 0.64 | 0.65 |
| | Median | 0.84 | 0.84 |
| | Max | 1.07 | 1.10 |
| Month 3 | Mean | 0.87 | 0.90 |
| | Min | 0.58 | 0.69 |
| | Median | 0.84 | 0.88 |
| | Max | 1.11 | 1.41 |
| Month 6 | Mean | 0.87 | 0.88 |
| | Min | 0.59 | 0.51 |
| | Median | 0.86 | 0.85 |
| | Max | 1.16 | 1.24 |
| Month 9 | Mean | 0.96 | 0.90 |
| | Min | 0.65 | 0.75 |
| | Median | 0.97 | 0.89 |
| | Max | 1.24 | 1.09 |
| Month 12 | Mean | 0.96 | 0.92 |
| | Min | 0.67 | 0.72 |
| | Median | 0.92 | 0.90 |
| | Max | 1.48 | 1.37 |

being the random effect and "visit number" and "treatment group" being the fixed effects. For the wheezing score and rescue usage outcomes, logarithmic transformations were necessary to meet the assumption of normality of the error. The data for these outcomes included both estimates of 95% confidence intervals for the medians transformed back to the original scale (Bland and Altman, 1996) for each treatment group at each time point. There were no statistical differences between "wheezing score" and "rescue usage" when comparing either the borage oil or corn oil groups. $FEV_1$ could not be transformed to meet the assumption of normality and therefore the medians and ranges are presented in Table IV. Once again, there were no differences between the two groups. Similarly, using an ANOVA model, wherein all subjects belong to the "control" or "borage oil" groups throughout (i.e. including baseline measurements), there were no significant differences in peak flow measurements between groups for either fixed months ($p = 0.0977$) or overall ($p = 0.1415$) (data not shown). We also note that there were no significant differences in any of the parameters at any time point between the borage oil and the control group with respect to the quality of life evaluations (data not shown). These included evaluation of (1) regular activities at home and at work; (2) social activities; (3) outdoor activities; (4) difficulty getting to sleep; (5) wake up during the night; (6) lack of a good night's sleep; (7) fatigue; (8) thirst; (9) reduced productivity; (10) tiredness; (11) poor concentration; (12) headache; (13) worn out; (14) inconvenience of having to carry tissues or handkerchief; (15) need to rub nose/eyes; (16) need to blow nose repeatedly; (17) stuffy/blocked; (18) runny; (19) sneezing; (20) post-nasal drip; (21) itchy eyes; (22) watery eyes; (23) sore eyes; (24) swollen eyes; (25) frustrated; (26) impatient or restless; (27) irritable; (28) embarrassed by your symptoms.

We also note that there were no differences in exhaled NO, further confirming the lack of clinical response (Table V). Our data would suggest that, although we can cause significant changes in biochemical features of the inflammatory response characteristic of asthma, we are not seeing a significant clinical response in clinical stable asthma.

TABLE V Quantitation of exhaled NO (in parts per billion)

| Treatment group | | Corn oil | Borage oil |
|---|---|---|---|
| Pre-Study | Median estimate | 28.7 | 22.0 |
| | 95% CI | (17.5, 47.1) | (15.7, 30.9) |
| Month 0 | Median estimate | 32.7 | 21.1 |
| | 95% CI | (18.8, 57.0) | (16.0, 27.9) |
| Month 3 | Median estimate | 28.3 | 23.5 |
| | 95% CI | (17.2, 46.6) | (17.4, 31.8) |
| Month 6 | Median estimate | 33.6 | 21.8 |
| | 95% CI | (14.5, 77.7) | (15.7, 30.2) |
| Month 9 | Median estimate | 37.4 | 24.2 |
| | Median estimate | (20.5, 68.2) | (17.2, 34.1) |
| Month 12 | Median estimate | 40.1 | 28.6 |
| | 95% CI | (16.7, 96.5) | (19.3, 42.4) |

### DISCUSSION

Overall, the results from this 12-month study of dietary supplementation of GLA-containing borage oil at a dose of 2.0 g GLA per day resulted in a marked *in vivo* elevation of DGLA (elongation metabolite of GLA) in patients PMN phospholipids. This increase also paralleled reduced PMN phospholipid AA and the suppression of $Ca^{2+}$ ionophore-induced *ex vivo* PMN generation of $LTB_4$. Taken together these results are consistent with reported findings in the epidermal phospholipids of normal guinea pigs fed GLA-containing borage oil (Miller and Ziboh, 1988; Miller *et al.*, 1990). Furthermore, dietary supplementation with GLA have also been reported to alter fatty acid profile and eicosanoids in healthy humans (Ziboh and Fletcher, 1992; Johnson *et al.*, 1997). Although shorter durations of dietary PUFAs had been reported, some of the findings have been conflicting. Since duration of dietary supplementation is essential in a nutritional study such as this, we were prompted to test our hypothesis for 12 months. This study underscores the duration of intake of the dietary supplement prior to the manifestation of alterations of the altered biochemical markers that were determined in this study. For instance, statistically significant increase in PMN-DLGA (elongation product of GLA) was revealed 6 months after the intake of GLA-containing borage oil implying that it would be prudent to conduct *in vivo* studies of this type for at least 6 months. Interestingly, the increase in PMN-DGLA continued to manifest itself 12 months after the intake of the GLA-containing borage oil (data not shown).

The elevation of PMN-DGLA paralleled the suppression of $Ca^{2+}$ ionophore-induced *ex vivo* generation of PMN-$LTB_4$. A typical relationship of PMN-DGLA increase and PMN-$LTB_4$ suppression after 12 months of borage oil ingestion is illustrated in Fig. 2. The data revealed that marked elevation of PMN-DGLA paralleled statistically significant suppression of PMN-$LTB_4$. The data clearly shows that a relationship exists between PMN accumulation of DGLA and the suppression PMN-$LTB_4$. What this study did not show is whether a dose of 2.0 g GLA/day was maximal for generating maximal PMN-DGLA for greater suppression PMN-generated $LTB_4$. Whether or not such a direct relation exists must await future dietary studies.

The mechanism by which dietary GLA dietary supplementation exerts its *in vivo* suppression of PMN generation of $LTB_4$ remains unclear. However, one possibility is that dietary GLA is converted *in vivo* by the elongase enzyme to DGLA. The resulting DGLA is metabolized via the 15-LOX to 15(*S*)-hydroxyeicosatrienoic acid (15-HETrE). Analysis for 15-HETrE in PMNs isolated from a small group of patients on dietary borage oil revealed elevated biosynthesis of PMN-15-HETrE when compared to the corn oil group (data not shown). Indeed, the *in vitro* transformation of DGLA to 15-HETrE has been reported in different systems (Miller *et al.*, 1988; Iversen *et al.*, 1991; Chilton *et al.*, 1996; Israel *et al.*, 1996). This metabolite (15-HETrE) has been reported to inhibit the *in vitro* transformation of AA to $LTB_4$ by rat basophilic leukemic (RBL) cells (Vanderhoek *et al.*, 1980; Miller *et al.*, 1988; Miller *et al.*, 1991) via 5-LOX. On the other hand, DGLA can also undergo oxygenation to generate prostaglandin $E_1$ ($PGE_1$) via the cyclooxygenase. This metabolite ($PGE_1$) has been reported to suppress acute chronic inflammation in adrenalectomized rats (Zurier *et al.*, 1973) and immune complex vasculitis in rats (Kunkel *et al.*, 1979), thus, implying that the accumulation of DGLA *in vivo* may function to generate two important *in vivo* metabolites (15-HETrE and $PGE_1$) that could attenuate the inflammation associated with clinical asthma. Overall, our biochemical findings imply that dietary supplementation with borage oil containing GLA can suppress *in vivo* neutrophil generation of pro-inflammatory $LTB_4$ and therefore can serve to prevent or attenuate the clinical course of asthma.

Leukotrienes (LT) are a family of potent lipid mediators of inflammation (Borgeat and Samuelsson, 1979; Murphy *et al.*, 1979) formed by the initial conversion of arachidonic acid (AA) to leukotriene $A_4$ ($LTA_4$) by the enzyme 5-LOX. $LTA_4$ is then converted either by $LTA_4$-hydrolase to leukotriene $B_4$ ($LTB_4$) or by glutathione transferase to the cysteinyl-leukotrienes (cys-LT), which include $LTC_4$, $LTD_4$ and $LTE_4$. While $LTB_4$ on the one hand, exhibits potent chemotactic activity in neutrophils and eosinophils (Smith *et al.*, 1980), the cys-LTs exhibit potent bronchoconstrictor activities (Piper *et al.*, 1980), thus, making the LTs a logical target for the treatment of a number of inflammatory diseases including asthma. Specific binding sites (receptors) for the cys-LTs have been identified in guinea pig and in human lung parenchyma (Kuehl *et al.*, 1984). In human bronchoprovocation studies, leukotriene $D_4$ ($LTD_4$) acts as a bronchospastic agent (Weiss *et al.*, 1983; Bisgaard *et al.*, 1985; Davidson *et al.*, 1987). More recently, a selective $LTD_4$/$LTE_4$-receptor antagonist has been found to result in significant improvement in FEV-1, wheezing and breathlessness (Cloud *et al.*, 1989). Importantly, Zileuton, a 5-LOX inhibitor, has been reported to attenuate asthma symptoms and to exert significant reduction in the incidence of exacerbations in patients with mild to moderate asthma (Israel *et al.*, 1996; Liu *et al.*, 1996). This promising effect is nonetheless dampened by reported elevation of liver enzymes in a sub-group of patients.

Dietary supplementation of GLA to patients with RA had clearly been reported to attenuate the signs and symptoms of inflammatory RA disease activity. Furthermore, dietary ingestion of GLA by normal human individuals did result in the suppression of the ability of their $Ca^{2+}$ ionophore activated PMNs to generate $LTB_4$ (Ziboh and Fletcher, 1992). Taken together, because 15-LOX metabolite of GLA/DGLA (15-HETrE) had been reported to inhibit $LTB_4$ generation by RBL-I cells *in vitro* (Vanderhoek *et al.*, 1980), we in this study tested the hypothesis that dietary supplementation of GLA-containing oil (borage oil) to mild and moderate asthmatic patients would result in elevated DGLA in the patients PMNs and result in the suppression of the asthmatic PMNs to biosynthesize $LTB_4$.

However, the data from our borage oil study are both biologically and clinically important. Although we have clearly demonstrated a meaningful change in leukotrienes, we have demonstrated that such a change is not accompanied by an improvement in asthma scores. This result has important specific implications for our understanding of biological mediators and asthma but also has generic implications for other inflammatory processes that have hypothesized that changes in leukotrienes mediated by diet will have clinical implications. Since asthma is a multifactorial disease process, it is likely that other contributory mediators to the pathogenesis of asthma were not responsive to inhibition of the 5-LOX pathway. Alternatively, the dose of gamma-linolenate (2 g/day) given to patients with mild to moderate asthma maybe inadequate to strongly inhibit all 5-LOX metabolites and thereby exert amelioration of the asthma. The power calculations that follow emphasize this point. The required pieces of information in any "power calculation" are the "detectable difference of interest", the "person-to-person" (or "between subject") and "within person, between observation" (or "residual") variations, and the desired power and size (often called "alpha level" or "level or significance"). As a rule, 80 and 5% are used for power and size, respectively, and that was used herein.

Regarding the "detectable difference of interest", we used the smallest difference between the groups that would be frequently referred to as "clinical" or "practical significance". This is because, if the actual effect is larger, the statistical tests will have greater-than-stated power (i.e. will be easier to detect); but, if the actual effect is smaller, it is less likely than the stated power that there will be statistical significance and these small differences would not be of clinical interest. As is always the case, a larger difference between the groups is easier to detect (i.e. would require a smaller sample size, all else being equal).

The estimates of variation come from our data sets and thus should be better than predictors prior to our study. Often, it is a good idea to increase the estimates of variation slightly to be conservative, as it is better to get a larger sample than is necessary than to have too small of a sample to be able to distinguish between the levels. We did not use that approach here, though. Rather we used exactly the variances observed in the data set. The reason that the person-to-person variation estimate is necessary is to allow overall comparisons between the two groups in the mixed model ANOVA that has been and will be used in the analysis for the data sets. To compare the two groups within any specific time period, however, the "within person, between observations" variations are used. Since baseline measurements are taken from both groups, the contrasts of greater interest will almost certainly be between the groups for a fixed time-point. For the clinical outcome "wheeze score", we used a logarithmic transformation in the analysis on the earlier data set. We added 1 to every observation before taking the natural logarithm of the value so as to include zeroes, a 25% decrease from the placebo group would actually be a difference of about 0.196 at the baseline means. Without the addition of 1 to the values, a 25% decrease from the placebo group is 0.2877, which is the value we used in the power calculations. Although one can propose the use of higher doses of borage oil for longer duration, the conclusion herein is that a significant biochemical alteration does not lead to a discernible clinical response. Asthma is clearly a multi-factorial process and dietary supplements, at least as used herein, do not appear to have clinical benefit.

Mixed model ANOVA methods were used to model the outcomes "PEF", "wheezing score" and "rescue usage", with the subjects being the random effect and "visit number" and "treatment group" being the fixed effects. For the wheezing score and rescue usage outcomes, logarithmic transformation were necessary to meet the assumption of normality of the error. The tables for these outcomes present estimates and 95% confidence intervals for the medians transformed back to the original scale (Bland and Altman, 1996) for each treatment group at each time point.

Overall, data from this study does demonstrate that dietary ingestion of gamma-linolenate as borage oil does elevate PMN DGLA and its 5-LOX metabolite 5-HETrE which parallel suppression of PMN $LTB_4$. Although our data did not reveal suppression of measured clinical scores, which likely may be due to other mediators contributing to the asthma (a multifactorial disease) or alternatively, the dose of 2.0 g GLA used may be inadequate to attenuate the multitude asthma processes. Nonetheless, these findings are interesting and warrant further explorations into the role of higher doses of GLA, $LTB_4$ and other possible mediators in asthma.

### Acknowledgements

The authors appreciate the helpful contribution of Marcy L. Crees in this project. Supported in part by NIH Research Grant AT000637 and NIH Research Grant 1P50AT00079-01 of the US Public Health Service.

### References

Bisgaard, H., Groth, S. and Madsen, F. (1985) "Bronchial hyperreactivity to leucotriene D4 and histamine in exogenous asthma", *Br. Med. J. (Clin. Res. Ed.)* **290**, 1468–1471.

Bland, J.M. and Altman, D.G. (1996) "Transformations, means, and confidence intervals", *Br. Med. J.* **312**, 1079.

Borgeat, P. and Samuelsson, B. (1979) "Transformation of arachidonic acid by rabbit polymorphonuclear leukocytes. Formation of a novel dihydroxyeicosatetraenoic acid", *J. Biol. Chem.* **254**, 2643–2646.

Borgeat, P., Hamberg, M. and Samuelsson, B. (1976) "Transformation of arachidonic acid and homo-gamma-linolenic acid by rabbit polymorphonuclear leukocytes. Monohydroxy acids from novel lipoxygenases", *J. Biol. Chem.* **251**, 7816–7820.

Boushey, H.A., Holtzman, M.J., Sheller, J.R. and Nadel, J.A. (1980) "Bronchial hyperreactivity", *Am. Rev. Respir. Dis.* **121**, 389–413.

Chilton, L., Surette, M.E., Swan, D.D., Fonteh, A.N., Johnson, M.M. and Chilton, F.H. (1996) "Metabolism of gammalinolenic acid in human neutrophils", *J. Immunol.* **156**, 2941–2947.

Chung, K.F. (1986) "Role of inflammation in the hyperreactivity of the airways in asthma", *Thorax* **41**, 657–662.

Cloud, M.L., Enas, G.C., Kemp, J., *et al.* (1989) "A specific LTD4/LTE4-receptor antagonist improves pulmonary function in patients with mild, chronic asthma", *Am. Rev. Respir. Dis.* **140**, 1336–1339.

Davidson, A.B., Lee, T.H., Scanlon, P.D., *et al.* (1987) "Bronchoconstrictor effects of leukotriene E4 in normal and asthmatic subjects", *Am. Rev. Respir. Dis.* **135**, 333–337.

Dunnill, M.S. (1960) "The pathology of asthma, with special reference to changes in the bronchial mucosa", *J. Clin. Pathol.* **13**, 27–33.

Glynn, A.A. and Michaels, L. (1960) "Bronchial biopsy in chronic bronchitis and asthma", *Istanb. Tip Fak. Mecm.* **15**, 142–153.

Israel, E., Cohn, J., Dube, L. and Drazen, J.M. (1996) "Effect of treatment with zileuton, a 5-lipoxygenase inhibitor, in patients with asthma. A randomized controlled trial. Zileuton Clinical Trial Group", *J. Am. Med. Assoc.* **275**, 931–936.

Iversen, L., Fogh, K., Bojesen, G. and Kragballe, K. (1991) "Linoleic acid and dihomogammalinolenic acid inhibit leukotriene B4 formation and stimulate the formation of their 15-lipoxygenase products by human neutrophils *in vitro*. Evidence of formation of antiinflammatory compounds", *Agents Actions* **33**, 286–291.

Johnson, M.M., Swan, D.D., Surette, M.E., *et al.* (1997) "Dietary supplementation with gamma-linolenic acid alters fatty acid content and eicosanoid production in healthy humans", *J. Nutr.* **127**, 1435–1444.

Kuehl, F.A. Jr., DeHaven, R.N. and Pong, S.S. (1984) "Lung tissue receptors for sulfidopeptide leukotrienes", *J. Allergy Clin. Immunol.* **74**, 378–381.

Kunkel, S.L., Thrall, R.S., Kunkel, R.G., McCormick, J.R., Ward, P.A. and Zurier, R.B. (1979) "Suppression of immune complex vasculitis in rats by prostaglandin", *J. Clin. Investig.* **64**, 1525–1529.

Lawson, L.D. and Hughes, B.G. (1988) "Triacylglycerol structure of plant and fungal oils containing gamma-linolenic acid", *Lipids* **23**, 313.

Leventhal, L.J., Boyce, E.G. and Zurier, R.B. (1993) "Treatment of rheumatoid arthritis with gammalinolenic acid", *Ann. Intern. Med.* **119**, 867–873.

Liu, M.C., Dube, L.M. and Lancaster, J. (1996) "Acute and chronic effects of a 5-lipoxygenase inhibitor in asthma: a 6-month randomized multicenter trial. Zileuton Study Group", *J. Allergy Clin. Immunol.* **98**, 859–871.

Miller, C.C. and Ziboh, V.A. (1988) "Gammalinolenic acid-enriched diet alters cutaneous eicosanoids", *Biochem. Biophys. Res. Commun.* **154**, 967–974.

Miller, C.C., McCreedy, C.A., Jones, A.D. and Ziboh, V.A. (1988) "Oxidative metabolism of dihomogammalinolenic acid by guinea pig epidermis: evidence of generation of anti-inflammatory products", *Prostaglandins* **35**, 917–938.

Miller, C.C., Ziboh, V.A., Wong, T. and Fletcher, M.P. (1990) "Dietary supplementation with oils rich in (*n*-3) and (*n*-6) fatty acids influences *in vivo* levels of epidermal lipoxygenase products in guinea pigs", *J. Nutr.* **120**, 36–44.

Miller, C.C., Tang, W., Ziboh, V.A. and Fletcher, M.P. (1991) "Dietary supplementation with ethyl ester concentrates of fish oil (*n*-3) and borage oil (*n*-6) polyunsaturated fatty acids induces epidermal generation of local putative anti-inflammatory metabolites", *J. Investig. Dermatol.* **96**, 98–103.

Murphy, R.C., Hammarstrom, S. and Samuelsson, B. (1979) "Leukotriene C: a slow-reacting substance from murine mastocytoma cells", *Proc. Natl Acad. Sci. USA* **76**, 4275–4279.

Piper, P.J., Samhoun, M.N., Tippins, J.R., Morris, H.R. and Taylor, G.W. (1980) "Slow-reacting substances and their formation by a lipoxygenase pathway", *Agents Actions* **10**, 541–547.

Recommendations for Standardized Procedures for the Online and Offline Measurement of Exhaled Lower Respiratory Nitric Oxide and Nasal Nitric Oxide in Adults and Children, 1999.

Smith, M.J., Ford-Hutchinson, A.W. and Bray, M.A. (1980) "Leukotriene B: a potential mediator of inflammation", *J. Pharm. Pharmacol.* **32**, 517–518.

Stone, K.J., Willis, A.L., Hart, W.M., Kirtland, S.J., Kernoff, P.B. and McNicol, G.P. (1979) "The metabolism of dihomo-gamma-linolenic acid in man", *Lipids* **14**, 174–180.

Vanderhoek, J.Y., Bryant, R.W. and Bailey, J.M. (1980) "Inhibition of leukotriene biosynthesis by the leukocyte product 15-hydroxy-5,8,11,13-eicosatetraenoic acid", *J. Biol. Chem.* **255**, 10064–10066.

Weiss, J.W., Drazen, J.M., McFadden, E.R. Jr., *et al.* (1983) "Airway constriction in normal humans produced by inhalation of leukotriene D. Potency, time course, and effect of aspirin therapy", *J. Am. Med. Assoc.* **249**, 2814–2817.

Ziboh, V.A. and Fletcher, M.P. (1992) "Dose-response effects of dietary gamma-linolenic acid-enriched oils on human polymorphonuclear-neutrophil biosynthesis of leukotriene B4", *Am. J. Clin. Nutr.* **55**, 39–45.

Ziboh, V.A., Yun, M., Hyde, D.M. and Giri, S.N. (1997) "Gamma-linolenic acid-containing diet attenuates bleomycin-induced lung fibrosis in hamsters", *Lipids* **32**, 759–767.

Zurier, R.B., Hoffstein, S. and Weissmann, G. (1973) "Suppression of acute and chronic inflammation in adrenalectomized rats by pharmacologic amounts of prostaglandins", *Arthritis Rheum.* **16**, 606–618.

Zurier, R.B., Rossetti, R.G., Jacobson, E.W., *et al.* (1996) "Gamma-linolenic acid treatment of rheumatoid arthritis. A randomized, placebo-controlled trial", *Arthritis Rheum.* **39**, 1808–1817.

